# 2164: Emotional dysfunction and stigma: Its effects on HIV-associated neurocognitive disorders (HAND)

**DOI:** 10.1017/cts.2017.163

**Published:** 2018-05-10

**Authors:** Michell R. Aponte, Maribella González-Viruet, Valerie Wojna

**Affiliations:** University of Puerto Rico-Medical Sciences Campus, San Juan, Puerto Rico

## Abstract

OBJECTIVES/SPECIFIC AIMS: HIV is a chronic disease that affects the immune system. HIV+ people live more thanks to effective antiretroviral treatments. The scientific data demonstrate that HIV+ is associated to the cognitive impairment presented in the 50% of the patient. The objective of this study is to determine the correlation between emotional dysfunction and perceived stigma in HIV+ women and its effects HIV-associated neurocognitive disorders (HAND). METHODS/STUDY POPULATION: HIV+ women will be recruited form the Hispanic Longitudinal Cohort and evaluated questionnaires for emotional dysfunction and stigma, neuropsychological tests, and MRI. RESULTS/ANTICIPATED RESULTS: We anticipated that women with HIV+ will experience higher levels of emotional dysfunction (ie, fear) and perceived stigma when compared with the control group. Women with HIV infection will present an association between emotional dysfunction most like fear and perceived stigma when compared with the control group. This correlation will be associated with HAND. The women with HIV infection will present circuit integrity dysfunction associated with emotional dysfunction and perceived stigma as determined by DTI and connectivity (MRI). DISCUSSION/SIGNIFICANCE OF IMPACT: HIV stigma and emotional dysfunction have a negative impact in quality of life (QOL). This effect can be improved with several treatment interventions with eventual improvement in adherence, emotional control, and QOL.

